# Isolation and genomic characterization of the plant growth-promoting rhizobacterium GZY0 and its biocontrol potential against soft rot in *Amorphophallus konjac*

**DOI:** 10.3389/fmicb.2025.1641541

**Published:** 2025-12-03

**Authors:** Yu Zhong, Juan Wu, Zhen Ren, Cheng Wu, Yingying Qian, Luoping Wang, Xu Chen, Lisha Niu, Zuoxin Tang, Yuyu Zhang, Yanhong Wang, Tiyuan Xia

**Affiliations:** 1 College of Agriculture and Life Sciences, Kunming University, Kunming, China; 2 Raw Material Center of China Tobacco Yunnan Industrial Co., Ltd., Kunming, China

**Keywords:** *Amorphophallus konjac*, soft rot, *Bacillus velezensis*, whole-genome sequencing, plant growth promotion, antagonism, biocontrol

## Abstract

Bacterial soft rot is a destructive disease that hinders the production of *Amorphophallus konjac*. In this study, a bacterial strain (GZY0) antagonistic to *Pectobacterium aroidearum* was isolated from the rhizospheric soil of *A. konjac* using the solid agar plate confrontation test. Through 16S rRNA sequencing, gyrA gene sequencing, and ANI analysis, this GZY0 strain was identified as *Bacillus velezensis*. The cell suspension and cell-free supernatant of GZY0 produced bacteriostatic inhibition zones with diameters of 15.63 and 16.70 mm, respectively, against *P. aroidearum*. Both *in vitro* antagonist assays and greenhouse pot experiments demonstrated that GZY0 is effective at controlling soft rot in *A. konjac*. During *in vitro* antagonistic tests in *A. konjac* tissues, mixed inoculation with the cell suspension or cell-free supernatant of GZY0 provided control efficacy of 47.56% and 45.79%, respectively, compared with the inoculation of a *P. aroidearum* bacterial suspension alone. Greenhouse pot experiments further validated its disease-preventing potential, the disease index of the GZY0 + *P. aroidearum*-coinoculated group was 45.81% lower than that of the *P. aroidearum*-inoculatedgroup. Additional functional studies revealed that strain GZY0 exhibits excellent plant growth-promoting properties and capable of producing phosphorus-solubilizing zones on both inorganic and organic phosphorus media, with diameters of 11.68 mm and 7.50 mm, respectively. On CAS iron-supplemented medium, GZY0 produces an orange-yellow halo with a diameter of 11.33 mm and an A/Ar ratio of 1.19. Furthermore, GZY0 turns Salkowski reagent red, with an IAA yield of 17.77 mg/L. Furthermore, GZY0 exhibited antagonistic effects against *Botryosphaeria dothidea, Fusarium oxysporum, F. solani, F. oxysporum f. sp. panax*, and *Colletotrichum gloeosporioides*. Whole-genome sequencing demonstrated that the genome length of GZY0 was 4053804 bp and included 3,934 coding genes. In order to elucidate the biocontrol mechanisms of strain GZY0, genes related to nitrogen fixation, phosphorus solubilization, and IAA and siderophore production were identified, and 12 antagonism-related secondary metabolite synthesis gene clusters and induced systemic resistance-related genes were predicted. A total of 5,169 pan-genes were detected in the comparative genome, all possessing open pan-genes and closed core genes. Additionally, 3,240 homologous genes were shared among the six *Bacillus* spp. strains, with GZY0 harboring 171 unique homologous genes. In summary, our findings demonstrated that GZY0 exhibits plant growth promotion capabilities and serves as a potential biocontrol agent for soft rot in *A. konjac*. Hence, this strain warrants further development and application.

## 1 Introduction

*Amorphophallus konjac*, a perennial herb belonging to the family Araceae Juss., is widely grown in Southeast Asia and Africa. *A. konjac* contains large amounts of konjac glucomannan (KGM), which is widely used in food products, health products, pharmaceuticals, cosmetics, and chemicals due to its superb thickening and film-forming properties (Devaraj et al., 2019). Soft rot disease in *A. konjac*, whichis primarily caused by bacterial pathogens such as *Pectobacterium* spp., *Dicyeya* spp., and *Erwinia* spp., affects *A. konjac* growth and results in reduced quality and yield (Gao et al., 2024). China is a major producer of *A. konjac* and has the largest *A. konjac* production capacity in the world (He, 2021). The bacterial soft rot in *A. konjac* caused by *P. aroidearum* is prevalent in Yunnan, Sichuan, Shanxi, and other major *A. konjac* production areas in China. Owing to the increasing market demand for *A. konjac*, the scale of *A. konjac* production is expanding, and large amounts of chemical fertilizers and pesticides are being applied to promote the growth of this plant. However, this has created continuous cropping challenges, seriously affecting the yield and quality of *A. konjac*. In this context, the high incidence of soft rot has emerged as a major hindrance to the development of the *A. konjac* industry (Zhang L. et al., 2022; Yang et al., 2023).

At present, the strategies aimed at soft rot prevention and control in *A. konjac* are focused on the integration of various breeding methods for selecting disease-resistant *A. konjac* strains with high economic value, and the development of agents that mitigate the impact of soft rot in this plant. Chemical compounds such as streptomycin, benzothiazolinone, flusilazole, and bromothalonil have shown a certain degree of efficacy in controlling the development of soft rot in *A. konjac*. However, the long-term use of chemical agents not only increases pathogen resistance but also generates pesticide residues in agricultural products, causing environmental pollution and thereby affecting the sustainable development of the *A. konjac* industry. Given the focus on green agriculture and increasing awareness regarding food safety, eco-friendly prevention and control strategies for soft rot in *A. konjac* have become imperative.

Recently, some researchers have screened antagonistic bacteria for the biocontrol of bacterial soft rot. Nevertheless, due to the influence of abiotic and biotic factors, existing biological agents and bio-organic fertilizers offer unpredictable effects during practical application (He, 2021). There have been few studies regarding the use of bacterial biocontrol agents for soft rot in *A. konjac*, the range of microbial agents available for practical production applications is relatively limited. Previous studies have focused on strain isolation and bacteriostatic activity analysis (Meng et al., 2025; Dai et al., 2021), with limited research on functional bacteria that can promote the growth of *A. konjac*, additionally, the biocontrol performance of these strains has never been explored at the genome level. Given that the rhizosphere micro-ecosystems of *A. konjac* plays a critical role in the development of soft rot and regulate the efficacy of microbial biocontrol agents, this study selected plant growth-promoting rhizobacteria (PGPR) isolated from the *A. konjac* rhizosphere to identify superior strains possessing both growth-promoting and biocontrol potential.

PGPR are the microorganisms that live within the rhizosphere of plants, directly or indirectly promoting plant growth. These rhizobacteria—including nitrogen-fixing bacteria, *Bacillus*, and *Agrobacterium*—serve as important resources for biocontrol. Among them, *Bacillus* spp. represent a class of gram-positive *Corynebacterium* that are widely distributed in the natural environment, are harmless to humans and animals, do not cause environmental pollution, and have strong adaptability to complex environments due to their endospore production capacity. *Bacillus* can produce antimicrobial peptides, bacteriocins, and other substances to antagonize pathogenic plant fungi and reduce the incidence of seedling disease. Furthermore, these bacteria can also secrete plant growth promotion (PGP) substances, enhancing the growth and development of seedlings. For instance, Dong et al. (2021) screened four *Bacillus* spp. antagonistic bacteria from the rhizosphere soil of Areca taro [*Colocasia esculenta* (L.) *Schott*], all of which demonstrated effective biocontrol of taro soft rot disease in field experiments. Cui et al. (2021) reported that both *B. velezensis* BPC16 and W2-7 suppressed the growth of *P. aroidearum* and reduced the incidence of soft rot in in *A. konjac*, with biocontrol efficacies of 43.01% and 31.99%, respectively.

After obtaining the relevant soil sampling permits, in this study, strain GZY0 was isolated from the rhizospheric soil of *A. konjac* growing in Reshui Town, Xuanwei County, Qujing City, Yunnan Province, China (26.0785N, 103.7729E), and was selected based on its strongest *in vitro* antagonistic activity against *P. aroidearum* among 59 isolates. GZY0 was identified as *B. velezensis*, and the effectiveness of GZY0 in controlling soft rot in *A. konjac* was studied *in vitro* and *in vivo* experiments, the PGP features of this strain were also explored. A genomic analysis of strain GZY0 was conducted, and genes related to antimicrobial activity, plant growth promotion, and ability to induce host systemic resistance, and the mechanism of metabolite synthesis was examined. This study provides technical and theoretical support for the application of the GZY0 strain in the prevention and treatment of soft rot in *A. konjac*.

## 2 Materials and methods

### 2.1 Plants, strains, and media

*A. konjac*: *A. konjac* (*A. konjac* K. Koch) was provided by Yunnan Jingtian Konjac Agriculture Co., Ltd.

Biocontrol strain: *B. velezensis* GZY0 was isolated and screened from the rhizosphere soil of *A. konjac* in Damogu Village, Luliang County, Qujing City, Yunnan Province (24.9361 N, 103.5809 E). The genome of this strain has been sequenced and uploaded to NCBI (SRP636943).

Pathogens: *P. aroidearum, Botryosphaeria dothidea, F. oxysporum, F. solani, F. oxysporum f. sp. panax*, and *Colletotrichum gloeosporioides* were provided by the Department of Plant Protection, Kunming University.

Culture media and reagents: LB solid medium (Haibo, China), LB liquid medium (Haibo, China), CAS solid medium (Haibo, China), Pikovskaya inorganic phosphorus medium (Nautiyal, 1999), and organic phosphorus solid medium were prepared as described previously (Nautiyal, 1999), with minor adjustments (10 g/L glucose, 2 g/L Phytin, 5 g/L MgCl_2_·6H_2_O, 0.25 g/L MgSO_4_·7H2O, 0.2 g/L KCl, 0.1 g/L (NH_4_)_2_SO_4_, and 15 g/L agar powder). Meanwhile, Salkowski reagent (Patten and Glick, 2002; Khan et al., 2019), the CAS detection solution (Louden et al., 2011), Ashby nitrogen-free medium (Abdallah et al., 2018), and MKB liquid medium were prepared as described by Wang et al. (2022), with minor adjustments (5.0 g of casamino acid, 15.0 mL of glycerol, 2.5 g of K_2_HPO_4_, and 0.2 g of MgSO_4_·7H_2_O; pH = 7.0).

Preparation of bacterial suspension: the target strain was cultured overnight on LB solid medium, transferred to sterile LB liquid medium, and incubated at 28 °C and 120 r/min for 48 h. The culture was centrifuged at 10,000 r/min for 5 min, and the cells were re-suspended in sterile water until the OD600 reached 0.3.

Preparation of cell-free supernatant (CFS): after overnight incubation on LB solid medium, the target strain was transferred to sterile LB liquid medium and incubated at 28 °C and 120 r/min for 48 h. The culture was centrifuged at 10,000 r/min for 5 min, and the supernatant was isolated. The CFS was obtained by filtering the aforementioned supernatant through a 0.22 μm sterile filter membrane. The sterility of the CFS was confirmed by plating 100 μL onto LB agar plates and observing no microbial growth after 72 h of incubation at 37 °C.

### 2.2 Isolation of GZY0 and *in vitro* antibacterial assays

A total of 2 g of soil sample was added to 18 mL of sterile water containing glass beads, and the suspension was shaken for 20 min at 120 r/min and 28 °C. The suspension was serially diluted to 10-2, 10-3, and 10-4 in sterile water, and 100 μL of each dilutions were plated onto LB solid agar and incubated at 28 °C for 1–2 d. The colonies were picked up and steaked separately onto LB solid agar. The purified isolates were stored at 4 °C for antibacterial activity assays. The plate confrontation method (Yu et al., 2011) was used to determine the antibacterial activity of the purified isolates against *P. aroidearum* with minor modifications. Specifically, 150 μL of *P. aroidearum* suspension was added to 150 mL of sterilized LB agar medium (pre-cooled to 50 °C), the mixture was thoroughly mixed and poured into sterile plate, and cooled at room temperature until fully solidified. A single colony of potential antagonistic bacteria was spotted on the LB plate containing *P. aroidearum*, and incubated at 28 °C for 2 d. The antagonistic activities against *P. aroidearum* were evaluated by measuring the diameter of the inhibition zone (mm) around the isolated bacteria. The candidate strain GZY0 with the best antagonistic activity against *P. aroidearum* was selected.

Holes (5 mm in diameter) were punched in the center of the plate using a sterile perforator, and 10 μL of bioprophylaxis sterile fermentation solution was added to the holes to determine the diameter of the ring of inhibition (Ma et al., 2023).

### 2.3 Identification of strain GZY0

#### 2.3.1 Morphological identification

Strain GZY0 was inoculated in LB solid medium and incubated at 28 °C for 24 h. The shape, size, color, and wetness of the colonies were observed. Gram staining was performed according to the manual for common bacterial identification (Dong and Cai, 2001).

#### 2.3.2 Molecular identification

Strain GZY0 was sent to Shenzhen BGI Genomics Co., Ltd. for 16s RNA (27FAGAGTTTGATCCTGGCTCAG, 1492RTACGGCTACCTTGTTACGACTT) and housekeeping gene gyrA (42FCAGTCAGGAAATGCGTACGTCCTT, 1066RCAAGGTAATGCTCCAGGCATTGCT) sequencing. The sequencing results were subjected to BLAST alignment in NCBI, and a phylogenetic tree was constructed for the strains with highest similarity to GZY0 using the neighbor-joining method in MEGA 7.0.26 software, with 1,000 bootstrap replications.

#### 2.3.3 Average nucleotide identity (ANI) analysis of the whole-genome sequence of GZY0 based on the model strain *B. velezensis* FZB42

ANI analysis allows the comparison of genetic relationships between microbial genomes at the nucleotide level. Generally, ANI values greater than 95% indicate that the microbes belong to the same species (Arahal, 2014), but Ciufo et al. (2018) increased this threshold from 95% to 96%. In this study, the ANI values of GZY0 vs. the model strain *B. velezensis* FZB42 were calculated using the online ANI calculator tool (http://enve-omics.ce.gatech.edu/ani/index).

### 2.4 Whole-genome sequencing, annotation, and comparative genomics

Strain GZY0 was inoculated in LB liquid medium, incubated at 28 °C and 120 r/min overnight, and centrifuged at 4 °C and 8,000 r/min for 10 min. The cells were collected in centrifuge tubes after discarding the supernatant and washed thrice with PBS before freezing in liquid nitrogen for 1 h. The tubes were placed on dry ice and sent to Shanghai Major Biomedical Technology Co., Ltd. for whole-genome sequencing using the second-generation and third-generation sequencing method of Illumina and BacBio.

Based on ANI analysis, five closely related *Bacillus* spp. strains with fully sequenced genomes, including *B. velezensis* FZB42, *B. velezensis* SQR9, *B. siamensis* KCTC 13613, *B. amyloliquefaciens* DSM 7, and *B. amyloliquefaciens* NRRL B-14393, were selected to genomic comparison using the Shanghai Majorbio Bio-pharm Technology Co., Ltd. cloud platform.

### 2.5 Biocontrol effect of the GZY0 strain on soft rot disease caused by *P. aroidearum*

To evaluate the effect of GZY0 cell suspension on soft rot disease caused by *P. aroidearum, A. konjac* tuber tissue inoculation was performed as described by Cui et al. (2021), with slight improvements. Healthy flowering konjac bulbs were taken and cut into 2 cm × 2 cm × 1.5 cm konjac bulb tissues, surface disinfected with 75% ethanol and sodium hypochlorite, and a 2.8 cm × 2.8 cm crisscross wound was made on the surface of the konjac block, inoculated with 20 μL of a 1:1 (v/v) mixture of *P. aroidearum* bacterial suspension (OD600 = 0.3) + GZY0 bacterial suspension, with 20 μL of Sterile water was used as blank control. Seven d later, the onset of the disease was observed and the disease index and control efficacy were counted (Hamaoka et al., 2021).

Grading standards are as follows. Level 0: konjac tuber is normal, no disease; Level 1: the disease area accounted for konjac tuber surface area of 1/8–1/4 (including 1/4); Level 2: the disease area accounted for konjac tuber surface area of 3/8–1/2 (including 1/2); Level 3: the disease area accounted for konjac tuber surface area of 5/8–3/4 (including 3/4); Level 4: the disease area accounted for konjac tuber surface area of the konjac tuber more than 7/8 (including 7/8).

Disease index = [∑(number of infections at each level × number of corresponding levels)]/(total number of investigations × number of the highest level) × 100%

Control efficacy = (control disease index – treatment disease index)/control disease index × 100%

To evaluate the effect of CFS of GZY0 on soft rot disease caused by *P. aroidearum*, a modified version of the method described by Li et al. (2019) was employed. *A. konjac* corm was cut in half, and its surface was perforated using a sterile perforator (diameter = 6 mm) and inoculated with 20 μL of a 1:1 (v/v) mixture of *P. aroidearum* bacterial suspension (OD600 = 0.3) + CFS of GZY0. The control treatment mixture was prepared using an equal amount of sterile LB medium instead of the GZY0 CFS. Sterile LB medium alone was used for the blank control. The incidence of soft rot was observed after 7 d, and the filter paper was kept moist with sterile water. The grading, disease index, and control efficacy were evaluated as described in Section 2.3.1.

### 2.6 Analysis of the PGP features of GZY0

For the nitrogen fixation test, 10 μL of the GZY0 bacterial suspension (OD600 = 0.3) was added to the center of an Ashby solid medium plate. The plate was inverted and incubated at 28–30 °C for 5 d to observe whether the bacterial colonies could grow normally (Wang et al., 2020; Wu et al., 2019).

For the phosphorus solubilization test, the GZY0 bacterial suspension (OD600 = 0.3) was inoculated onto a perforated (6 mm) organic phosphorus and inorganic phosphorus medium and incubated at 28 °C for 5 d. The presence of transparent rings was observed, and their diameter was measured.

For the siderophore assay, the GZY0 bacterial suspension (OD600 = 0.3) was inoculated onto CAS culture medium, and the presence of an orange halo was observed (Milagres et al., 1999). If such a halo was detected, quantitative analysis was carried out, as described previously (Machuca and Milagres, 2003).

To determine the IAA-producing ability of GZY0, the colorimetric Salkowski reagent method was used (Glickmann and Dessaux, 1995). If the test solution turned pink, quantitative analysis was performed. IAA standard solutions of 0 mg/L, 10 mg/L, 20 mg/L, 30 mg/L, 40 mg/L, and 50 mg/L were prepared, and their absorbance values were measured at 530 nm (OD530). The standard curve (y = 0.0069x + 0.0057, R2 = 0.9988) was plotted with the OD530 value along the vertical axis and the IAA concentration along the horizontal axis (Supplementary Figure S1). The IAA yield of strain GZY0 was calculated based on this standard curve.

### 2.7 Inhibitory activity of GZY0 against different plant pathogens

The inhibitory effect of strain GZY0 against different pathogenic fungi was determined using the plate confrontation test. Preserved pathogenic strains were inoculated onto PDA plates for activation, while GZY0 was inoculated on LB solid medium. Strain GZY0 and pathogenic fungi were simultaneously inoculated on PDA plates, with the pathogenic fungi inoculated at the center of the plate. The diameter of the fungal plugs was 6 mm, and strain GZY0 was inoculated at an equal distance of 2.5 cm from the center of the plug using a toothpick, with three replicates per dish. The plates were incubated at 28 °C, and the fungistatic rate of strain GZY0 against the pathogens was determined based on fungal growth:

Fungistatic rate = [(control colony diameter – treatment colony diameter)/control colony diameter] × 100%.

### 2.8 Pot-based control efficacy test of GZY0 against *P. aroidearum*

Healthy *A. konjac* seedballs of uniform size were selected and planted in sterilized plastic pots with a base diameter of 7.6 cm, height of 9 cm, and inner diameter of 10 cm, with one plant in each pot. The planting substrate consisted of sterilized soil, organic soil, and vermiculite at a 1:2:2 ratio. There were three groups in total, with 15 pots per group, yielding a total of 45 pots. Cultivated in a greenhouse maintained at 28 °C and 75% relative humidity, the roots were irrigated with the GZY0 bacterial suspension (OD600 = 0.3) (50 mL per pot) once every 3 d for a total of three rounds. Control plants were treated with an equivalent amount of water instead of GZAY0. Three days after the irrigations were completed, wounds were induced on each A. konjac corm using a sterile syringe, and 50 mL *P. aroidearum* bacterial suspension (OD600 = 0.3) was added for root irrigation and inoculation. After inoculation, normal water and fertilizer management were administered. After 30 d of growth, the corm was removed, and the disease index and control efficacy were calculated as described in Section 2.5.

Grading standards are as follows. Level 0: no disease; Level 1: the onset of the area of the surface area of the konjac bulb 0%−20%; Level 2: the onset of the area of the surface area of the konjac bulb of 21%−40%; Level 3: the onset of the area of the surface area of the konjac bulb of 41%−60%; Level 4: the onset of the area of the surface area of the konjac bulb 61%−80%; Level 5: the onset of the area of the surface area of the konjac bulb of 81% and more than the konjac bulb. and above.

### 2.9 Statistical analysis

All the above experiments were repeated at least three times, and the results were expressed as the mean ± standard deviation (SD). All data were processed using Microsoft Excel 2021 and analyzed based on one-way analysis of variance (ANOVA) using IBM Statistics SPSS 27.0. Differences between groups were evaluated with Duncan's test, and a *P*-value of < 0.05 was considered statistically significant. The results of whole-genome sequencing were analyzed by the Major BioCloud platform.

## 3 Results

### 3.1 Identification and characterization of the GZY0 strain

**Figure 1:**
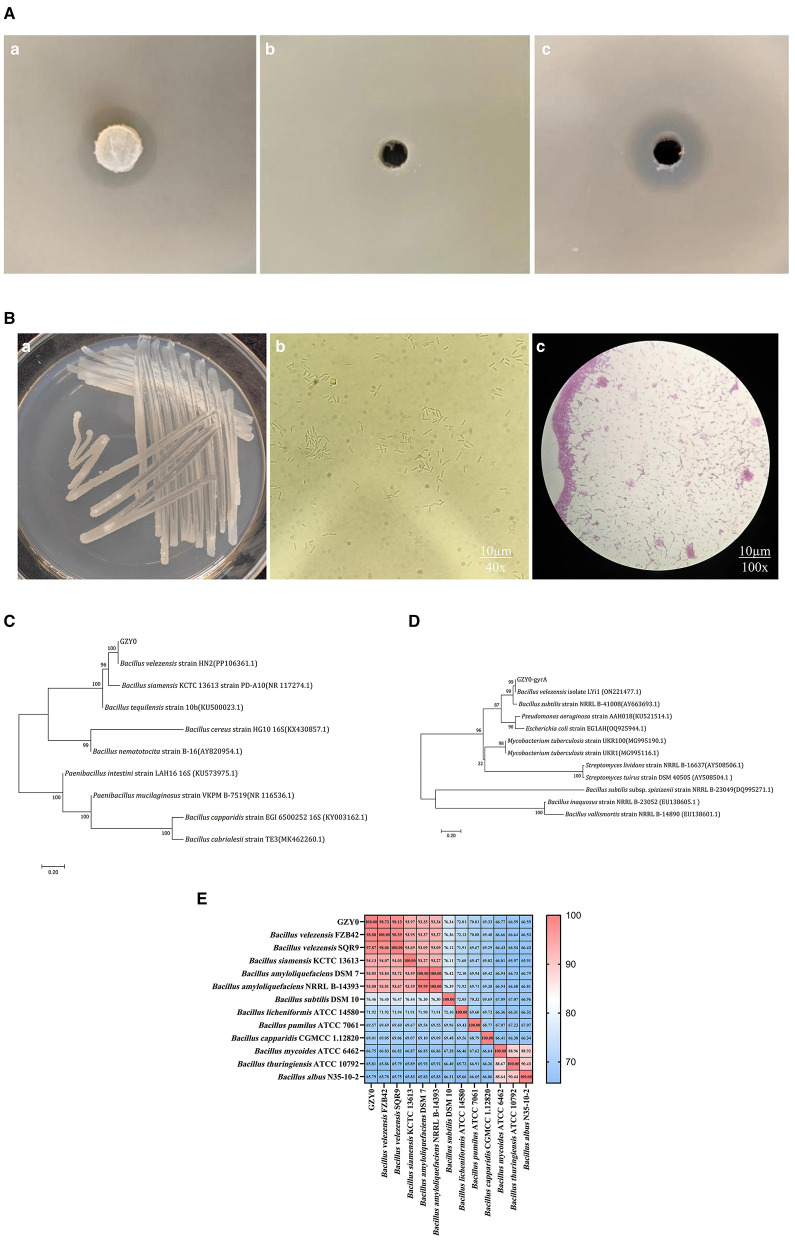
Characterization and identification of the GZY0 strain. **(Aa)** Antagonistic effect of strain GZY0 cell suspension against *P. aroidearum*. **(Ab)** Antagonistic effect of LB liquid medium against *P. aroidearum* (control). **(Ac)** Antagonistic effect of CFS of strain GZY0 on *P. aroidearum*. **(Ba)** Morphological characterization of strain GZY0. **(Bb)** Spore morphology. **(Bc)** Gram staining. **(C)** 16S rRNA phylogenetic tree of strain GZY0. **(D)** Phylogenetic tree of strain GZY0 housekeeping gene gyrA. **(E)** Heatmap of ANI values based on genome sequences of GZY0 and *Bacillus* spp. strains.

A total of 59 strains were isolated and purified from the rhizospheric soil of *A. konjac*. One strain with significant inhibitory effects against the pathogen causing soft rot in *A. konjac* was selected after primary and secondary screening and named GZY0. Compared with the control (Figure 1Ab), both the cell suspension and CFS of GZY0 showed obvious inhibitory effects against *P. aroidearum*, producing zones of inhibition with diameters of 15.63 mm (Figure 1Aa) and 16.70 mm (Figure 1Ac), respectively.

Strain GZY0 exhibits milky-white, opaque, and smooth colonies on LB agar plates and rod-shaped cells with oval spores under microscopy. Furthermore, strain GZY0 was identified as a Gram-positive bacterium (Figure 1B). The 16S rRNA and *gyrA* sequences of GZY0 were subjected to BLAST alignment (NCBI), and their lengths were 1,423 and 935 bp, respectively. The similarity between the 16S rRNA sequences of strain GZY0 and *B. velezensis* HN2 (PP106361.1) was 99.86% (Figure 1C). Similarly, the similarity between the housekeeping gene *gyrA* sequences of strain GZY0 and the *B. velezensis* isolate LYi1 (ON221477.1) was 100% (Figure 1D). Accordingly, strain GZY0 was identified as a strain of *B. velezensis*.

GZY0 had the highest ANI value of 98.88% with *B. velezensis* FZB42 and the second highest ANI value of 97.87% with *B. velezensis* SQR9. The ANI values of GZY0 with the above two *Bacillus* species were higher than 96%, and the ANI values of GZY0 with the other *Bacillus* species were 65.79%−94.13%. This further confirmed that strain GZY0 belonged to *B. velezensis* (Figure 1E).

### 3.2 Biological control effect of the GZY0 strain against *P. aroidearum*

**Figure 2:**
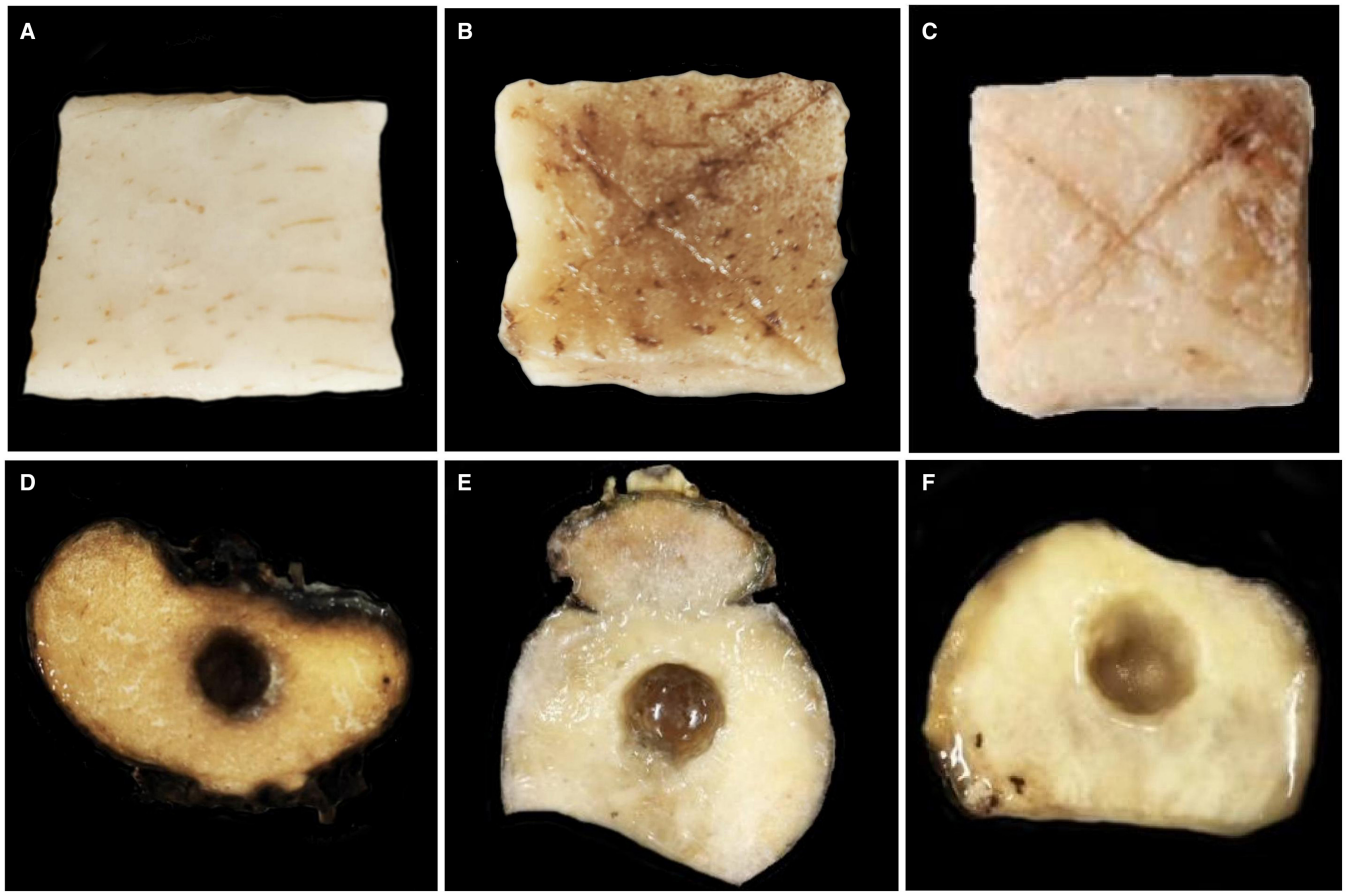
Biological control effect of the GZY0 strain against *P. aroidearum*. **(A)** Sterile water 20 μL. **(B)** *P. aroidearum* cell suspension: sterile water 1:1 mixture 20 μL. **(C)** *P. aroidearum* cell suspension: GZY0 cell suspension 1:1 mixture 20 μL. **(D)** LB liquid medium 20 μL. **(E)** *P. aroidearum* cell suspension: LB 1:1 mixture 20 μL. **(F)** *P. aroidearum* cell suspension: GZY0 cell-free supernatant 1:1 mixture 20 μL.

The *A. konjac* tuber tissue inoculation test revealed that the bacterial suspension and CFS of GZY0 exerted good *in vitro* antagonistic effects against soft rot in *A. konjac*. Compared to the control (Figures 2A, D), after 7 d of inoculation with *P. aroidearum* alone, the tuber tissue of *A. konjac* showed obvious morbidity and strong malodor (Figures 2B, E). However, after mixed inoculation with the GZY0 bacterial suspension and the CFS, tuber morbidity was significantly reduced (Figures 2C, F), and there was no bad odor; the control efficacy was 47.56% and 45.79%, respectively (Table 1).

**Table 1:** Control effect of GZY0 on *P. aroidearum in vitro* experiment.

**Treatment**	**Disease index**
A	0.00 ± 0.00 e
B	83.33 ± 4.17 b
C	43.70 ± 1.28 d
D	0.00 ± 0.00 e
E	84.72 ± 2.41 a
F	45.93 ± 0.43 c

(A) Sterile water 20 μL. (B) *P. aroidearum* cell suspension: sterile water 1:1 mixture 20 μL. (C) *P. aroidearum* cell suspension: GZY0 cell suspension 1:1 mixture 20 μL. (D) LB liquid medium 20 μL. (E) *P. aroidearum* cell suspension: LB 1:1 mixture 20 μL. (F) *P. aroidearum* cell suspension: GZY0 cell-free supernatant 1:1 mixture 20 μL. Data are means ± standard error, and different small letters indicate significant differences at the 0.05 level.

### 3.3 The PGP feature and the bacteriostatic activity of strain GZY0

**Figure 3:**
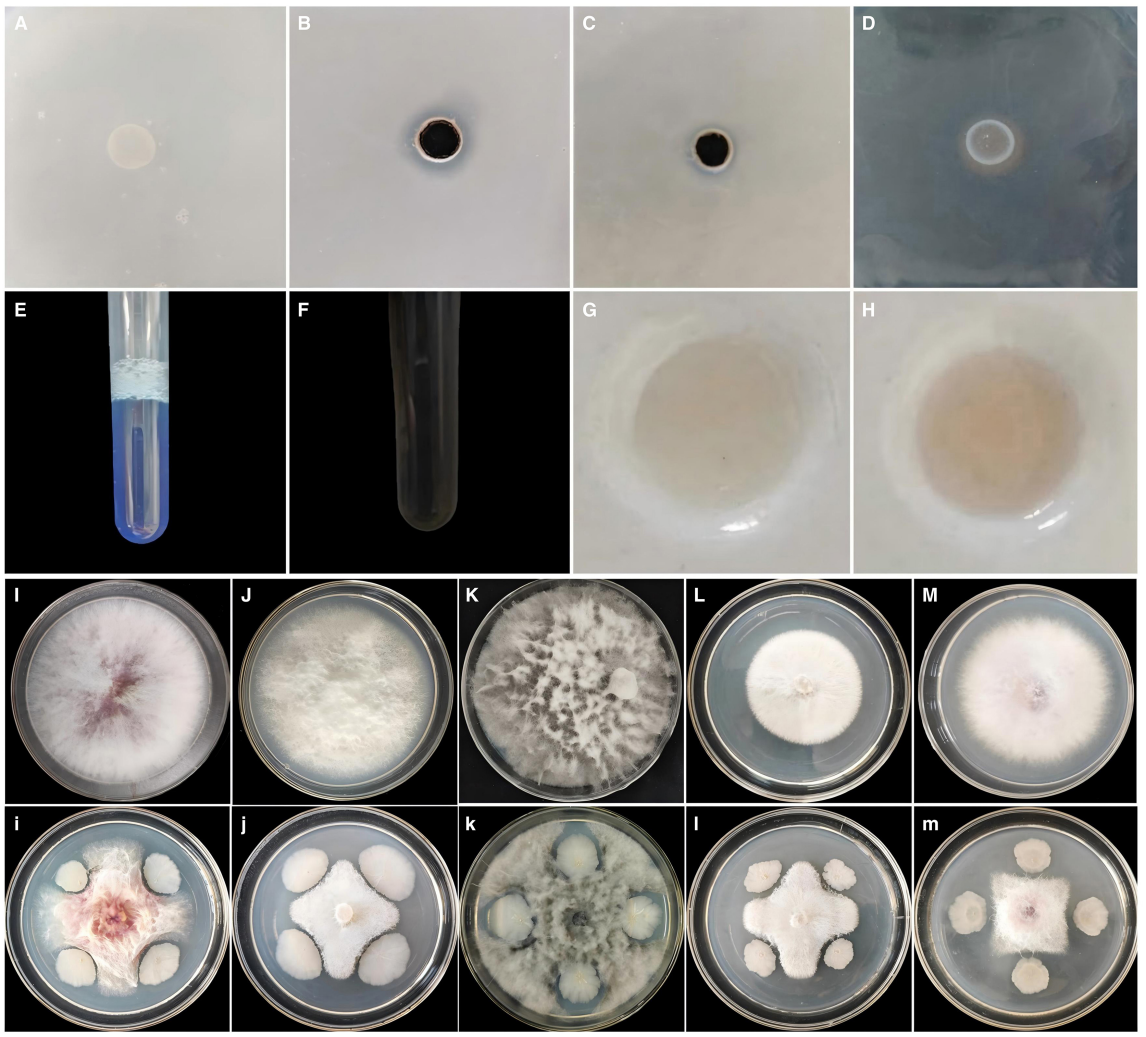
The PGP feature and the bacteriostatic activity of strain GZY0. **(A)** Nitrogen fixation effect of strain GZY0. **(B)** Dissolution of inorganic phosphorus by strain GZY0. **(C)** Dissolution of organic phosphorus by strain GZY0. **(D)** Qualitative determination of iron producing carriers of strain GZY0. **(E)** Quantitative determination of the ability of strain GZY0 to produce iron carriers (control). **(F)** Quantitative determination of the iron-carrier-producing ability of strain GZY0. **(G)** Qualitative determination of IAA-producing ability of strain GZY0 (control). **(H)** Qualitative determination of IAA-producing ability of strain GZY0. **(I, i)** *C. gloeosporioides*. **(J, j)** *F. solani*. **(K, k)** *B. dothidea*. **(L, l)** *F. oxysporum* f. *sp. panax*. **(M, m)** *F. oxysporum*. Uppercase letters are for control group and lowercase letters are for treatment group.

Strain GZY0 could grow on nitrogen fixation medium (Figure 3A), which indicated that it could fix nitrogen. GZY0 also produced a transparent phosphorus halo on inorganic phosphorus and organic phosphorus media [11.68 mm (Figure 3B) and 7.50 mm (Figure 3C), respectively], which suggested that it had the ability to dissolve both inorganic and organic phosphorus.

GZY0 produced an orange-yellow halo with a diameter of 11.33 mm on CAS iron-loaded medium (Figure 3D), demonstrating its siderophore production capacity. Its A/Ar ratio was found to be 1.19 (Figures 3E, F). Additionally, the GZY0 suspension turned red following the addition of Salkowski's reagent (Figures 3G, H), which suggested that this GZY0 can produce IAA. The IAA yield of strain GZY0 was quantitatively determined to be 17.77 mg/L.

In plate confrontation tests involving different pathogens, GZY0 exhibited fungistatic activity against *C. gloeosporioides, F. solani, B. dothidea, F. oxysporum f. sp. panax*, and *F. oxysporum*, with fungistatic rates of 30–55% (Table 2). Notably, compared to the control group (Figures 3I–M), GZY0 exhibits varying inhibition rates against different pathogens (Figure 3i–m), with the strongest fungistatic activity against *B. dothidea*, with fungistatic rates up to 53.17% (Figure 3k). The weakest fungistatic activity was against *F. solani* (30.36%) (Figure 3j).

**Table 2:** Determination of inhibitory effect of strain GZY0 on various plant pathogens.

**Pathogenic fungus**	**Inhibitory rate**
*C. gloeosporioides*	43.63 ± 1.98 b
*F. solani*	30.36 ± 1.22 d
*B. dothidea*	53.17 ± 1.34 a
*F. oxysporum f. sp. panax*	38.46 ± 2.04 c
*F. oxysporum*	36.47 ± 1.05 c

Data are means ± standard error, and different small letters indicate significant differences at the 0.05 level.

### 3.4 General genomic features of strain GZY0

**Figure 4:**
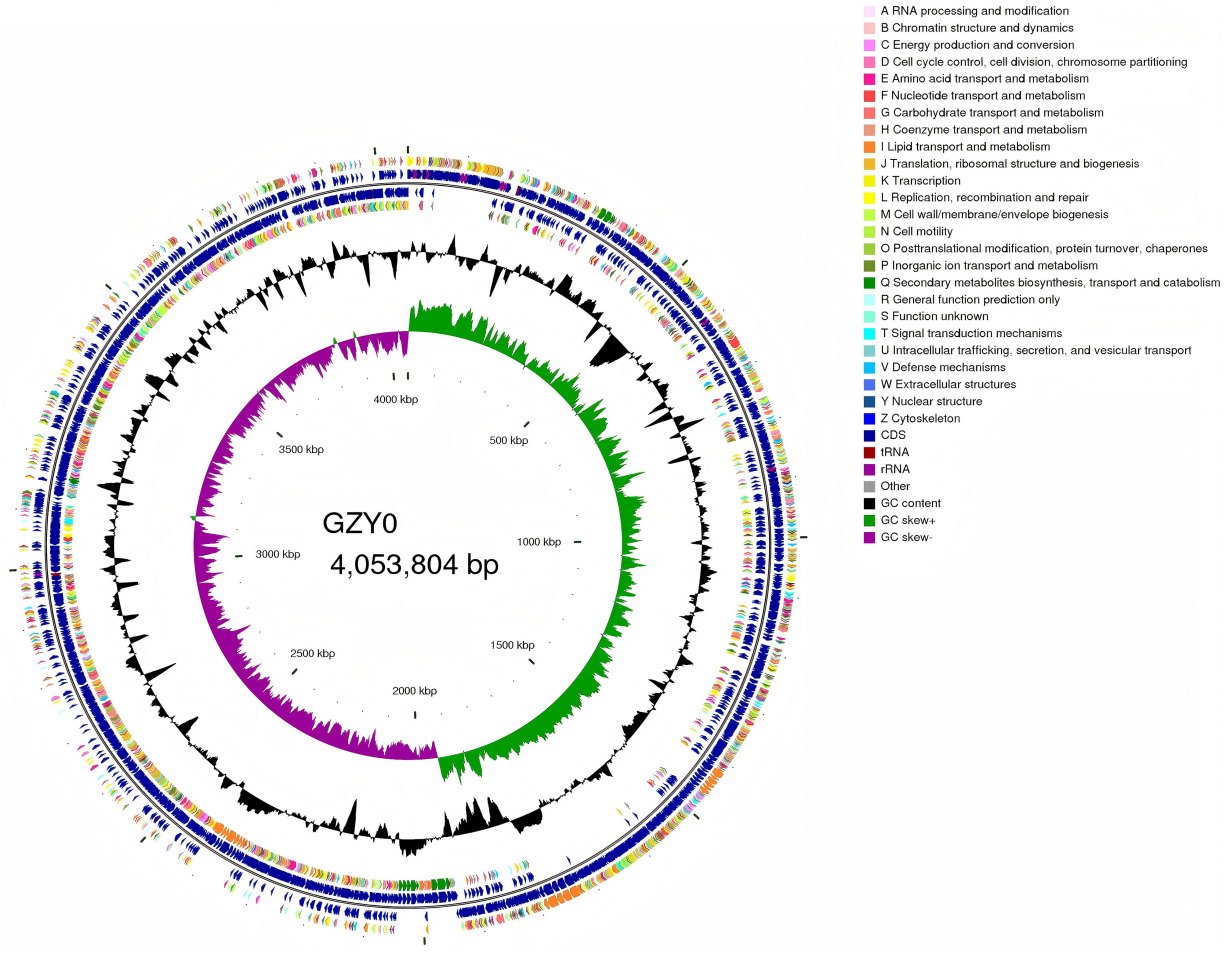
CGView genome circle map. The circle map from outside to inside the first and fourth circle for CDS on positive and negative strand, the second and third circle for CDS, tRNA, rRNA on positive and negative strand, respectively, the fifth circle for GC content, the sixth circle for GC-Skew value, and the innermost circle for the genome size identifier.

Using the unicycler assembly software, the GZY0 genome was assembled into a single circular chromosome with a total length of 4053804 bp and a sequencing depth of 349.84. The completeness score of 99.51% assessed by CheckM indicates that the assembly of GZY0 exhibits high integrity. Its G+C content was 46.53%, of which the A, T, G, and C contents were 1084228 bp, 1083494 bp, 943447 bp, and 942635 bp, respectively. A total of 3,934 coded genes were identified, with a total length of 3590091 bp, for one chromosome. Subsequently, tRNA and rRNA analyses showed that GZY0 contained 86 tRNAs and 27 rRNAs (Figure 4). A total of 130 genes were annotated through the CAZy database, and the number of genes enriched for glycoside hydrolases was 42, higher than that for other categories. Glycosyl transferases and carbohydrate esterases were linked to 41 and 32 genes, respectively (Supplementary Figure S2A).

In order to further analyze the genome of GZY0, sequencing data from GZY0 were aligned to the NR, COG, GO, SwissProt, KEGG, and Pfam databases. A total of 2,142 genes were annotated across the six databases, among which 3,928 genes were unique to the NR database, 3,168 genes to the COG database, 1,676 to the GO database, 3,602 to the SwissProt database, 2,898 to the KEGG database, and 3,458 to the Pfam database. Analysis via the COG database revealed that the genes were mainly involved in four groups of processes, including amino acid transport and metabolism, transcription, carbohydrate transport and metabolism, and general function only. Herein, amino acid transport and metabolism was linked to 311 genes; transcription, 300 genes; carbohydrate transport and metabolism, 287 genes; and general function only, 258 genes (Supplementary Figure S2B). Additionally, 807 genes were predicted in the PHI database using the Diamond software.

The GO database describes which biological, molecular functions, and cellular environments specific coding genes are involved in. The GO database contains three modules—Biological Process, Molecular Function, and Cellular Component. In this study, at the first level of classification, the highest number of genes from GZY0 were annotated to the Molecular Function module, followed by the Biological Process module. Among the secondary classifications, the highest number of genes were enriched for “membrane” in the Cellular Component module (345 genes). In the Biological Process module, the number of genes enriched for translation and phosphorylation (59 and 55 genes, respectively) was the highest. Finally, in the Molecular Function module, 228 and 188 genes were enriched for ATP binding and DNA binding, respectively. The results of GO analysis were important for subsequent genome function mining analysis (Supplementary Figure S2C). KEGG analysis showed that Metabolism was linked to the highest number of genes (i.e., 2,204) at the first level classification. Among these genes, 857 were involved in global and overview maps, accounting for the highest proportion of all secondary classifications. Carbohydrate metabolism was also enriched for a high number of genes (285 genes), while 229 genes were annotated to Amino Acid Metabolism and 216 genes were annotated to Metabolism of Cofactors and Vitamin. Hence, these three metabolic pathways likely played an important role in the antimicrobial function of GZA0 (Supplementary Figure S2D).

### 3.5 Mining for functional genes potentially associated with plant–bacteria interactions and antagonism

In order to further understand the PGP mechanism of strain GZY0, whole-genome sequencing was performed and genes related to PGP function were identified. The results showed that genes regulating the biosynthesis of IAA compounds (e.g., *trpA, trpB*, and *trpC*) were presented in the GZY0 genome and were involved in the synthesis of plant hormones. We hypothesized that GZY0 could directly promote plant growth and development by synthesizing IAA. GZY0 contained a variety of functional genes directly related to PGP (e.g., *glnB, trkH*, and *trkA*) that are involved in the synthesis of nitrogen and phosphorus, which are required for plant growth and development. We speculated that GZY0 may promote plant growth by improving the soil environment through nitrogen fixation and phosphorus solubilization. This strain contained some functional genes (e.g., *yclQ* and *fetB*) involved in the synthesis and transport of siderophores, which could increase the iron content in plants via iron chelation. The results indicated that these PGP-related genes in the genome of GZY0 may directly or indirectly promote plant growth (Supplementary Table S1).

The GZY0 genome also contained key genes promoting and inducing plant resistance. This included the functional genes *lytE* and *ykfC*, which regulate ABA biosynthesis. ABA can induce resistance to abiotic stress in plants and regulate physiological adaptation to abiotic stress. Additionally, we also identified key genes involved in the production of salicylic acid (*pheT* and *pheS*), which acts as a signaling molecule in the plant response to stress and can regulate important physiological and metabolic activities, strengthen the plant antioxidant defense system, and improve the stress resistance of plants (Supplementary Table S1).

The secondary metabolite synthesis gene clusters of GZY0 were predicted using antiSMASH. In total, 12 secondary metabolite synthesis gene clusters were predicted (Table 3), includes 3 NRPS, 1 RRE-containing, 1 PKS-like, 2 terpenes, 3 transAT-PKS, 1 T3PKS, and other types, and clear information was available for eight of them. Cluster1 (surfactin) contained 41 coding genes and shared 91% similarity with known gene clusters. Meanwhile, cluster2 (plantazolicin), cluster5 (macrolactin H), and cluster6 (bacillaene) contained 20, 44, and 44 coding genes and shared 91%, 100%, and 100% similarity with known gene clusters, respectively. Additionally, cluster7 (fengycin), cluster10 (difficidin), cluster11 (bacillibactin), and cluster12 (bacilysin) contained 66, 39, 45, and 42 coding genes, respectively, and shared 100% similarity with known gene clusters.

**Table 3:** Statistics of secondary metabolite synthesis gene cluster analysis.

**Cluster ID**	**Type**	**Similar cluster**	**Similarity (%)**	**Most similar known cluster**	**Gene no**.
Cluster1	NRPS	Surfactin	91	*B. velezensis* FZB42	41
Cluster2	RRE-containing	Plantazolicin	91	*B. velezensis* FZB42	20
Cluster3	PKS-like	Butirosin A/butirosin B	7	*B. circulans*	41
Cluster4	Terpene	–	–	–	22
Cluster5	TransAT-PKS	Macrolactin H	100	*B. velezensis* FZB42	44
Cluster6	TransAT-PKS	Bacillaene	100	*B. velezensis* FZB42	44
Cluster7	NRPS	Fengycin	100	*B. velezensis* FZB42	66
Cluster8	Terpene	–	–	–	22
Cluster9	T3PKS	–	–	–	49
Cluster10	TransAT-PKS	Difficidin	100	*B. velezensis* FZB42	39
Cluster11	NRPS	Bacillibactin	100	*B. subtilis* subsp. *subtilis str*. 168	45
Cluster12	Other	Bacilysin	100	*B. velezensis* FZB42	42

However, cluster3 (butirosin A/butirosin B), which contained 41 coding genes, only shared 7% similarity with known gene clusters. This suggested that this cluster in GZY0 may synthesize novel bacteriostatic substances and warrants further investigation. Meanwhile, three unknown secondary metabolite synthesis gene clusters (clusters 4, 8, 9) were detected in GZY0, of which two were linked to terpenes and one was linked to T3PKS. This showed that GZY0 may contain new antagonistic substance-related gene clusters. Additionally, these gene clusters comprise biosynthetic-additional, transport, regulatory, biosynthetic, and other genes (Supplementary Figure S3). This suggested that GZY0 could synthesize unique antibacterial substances to inhibit the growth of pathogens and indirectly promote plant growth through functional genes regulating the production of antimicrobial-related substances. Thus, GZY0 may have great potential applications in agricultural production.

### 3.6 Comparison of the GZY0 genome with other completely sequenced *Bacillus* spp.

**Figure 5:**
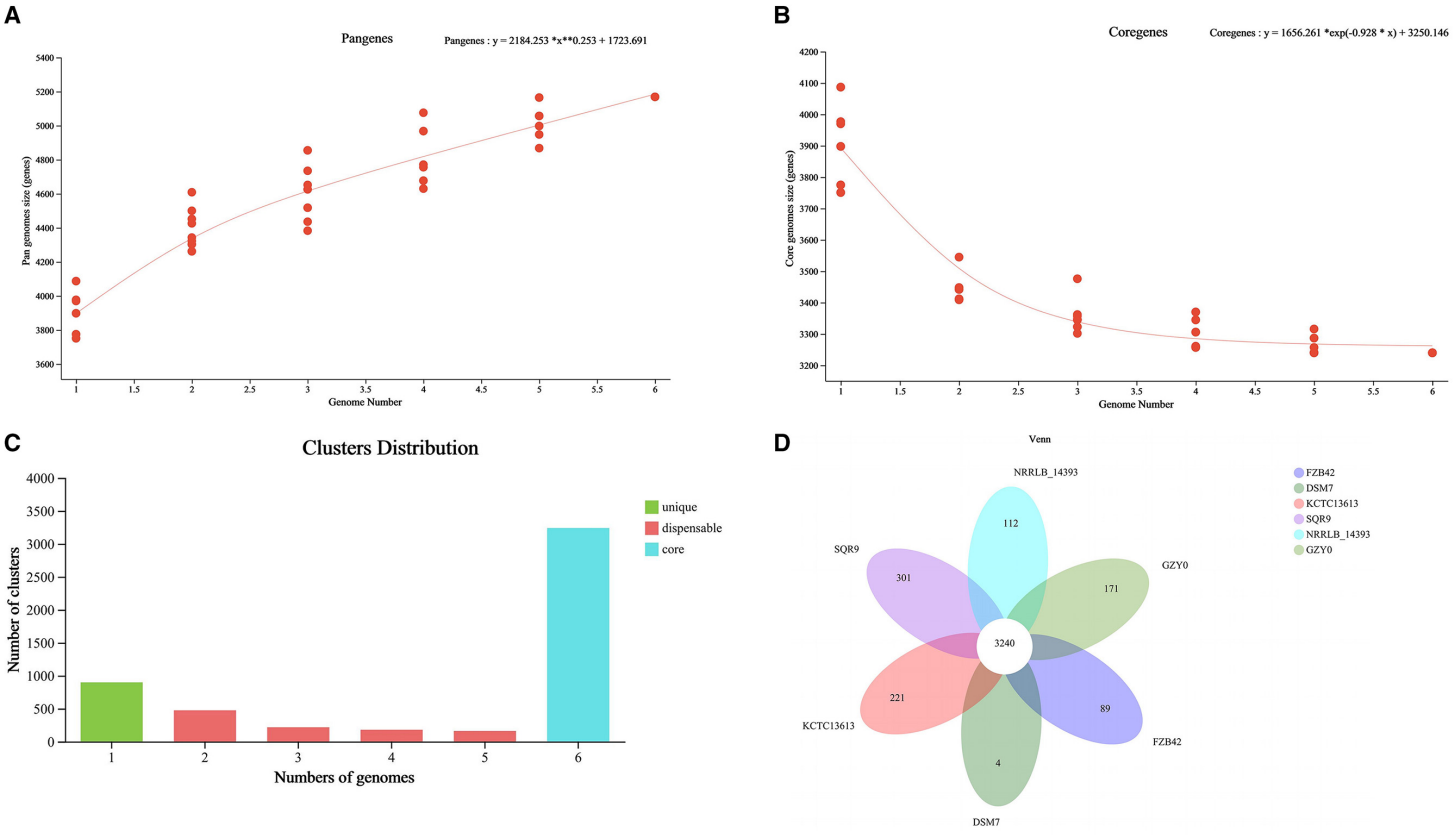
Comparison of GZY0 genome sequence against other five *Bacillus* spp. genomesequences. **(A)** Pangenome accumulation curve. **(B)** Core genome accumulation curve. **(C)** Statistical distribution of homologous genes across the genome. **(D)** Pangenome Venn diagram.

Pangenome analysis of GZY0 and five reference strains using Orthofinder software yielded a total of 5,169 pan-genome entities, including 3,240 core genes accounting for 62.68%. The pan-genome size formula is y = 2,184.253 ^*^ x^**^0.253 + 1,723.691. The parameter B (0.253) satisfies 0 < B < 1, indicating an open pan-genome. This suggests rich genetic diversity and the ability to rapidly adapt to environments by acquiring new genes (Figure 5A). The core gene formula is y = 1,656.261 ^*^ exp(−0.928 ^*^ x) + 3,250.146, where parameter B (−0.928) < 0. This indicates a closed-type core genome, suggesting all members share a stable genetic foundation (Figure 5B). Among the six *Bacillus* spp. strains, the number of core gene clusters was highest, followed by unique gene clusters, indicating genetic diversity and adaptive potential among strains (Figure 5C, Table 4). The six *Bacillus* spp. harbored 3,240 shared homologous genes. *B. velezensis* SQR9 possessed the highest number of unique homologous genes (301), while *B. amyloliquefaciens* DSM 7 had the fewest (4). GZY0 contained 171 unique homologous genes, suggesting it harbors numerous unique functions awaiting exploration (Figure 5D).

**Table 4:** Pangenome classification statistics table.

**Samples name**	**Total gene**	**Core**	**Dispensable**	**Unique**
GZY0	3,934	3,251	512	171
*B. velezensis* FZB42	3,766	3,248	429	89
*B. amyloliquefaciens* DSM 7	4,027	3,245	778	4
*B. siamensis* KCTC 13613	3,791	3,248	322	221
*B. velezensis* SQR9	3,993	3,254	438	301
*B. amyloliquefaciens* NRRL B-14393	4,313	3,347	854	112

### 3.7 The control efficacy of GZY0 against *P. aroidearum* in greenhouse

**Figure 6:**
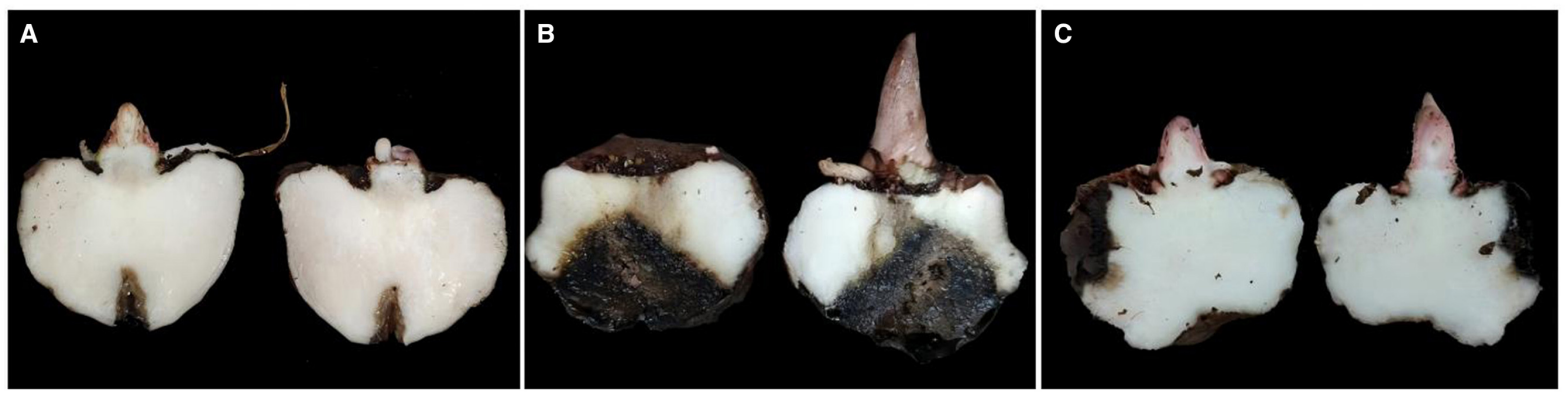
Determination of potting efficacy of strain GZY0 against *P. aroidearum*. **(A)** Root irrigation with water. **(B)** *P. aroidearum* cell suspension root irrigation. **(C)** GZY0 cell suspension + *P. aroidearum* cell suspension root irrigation (OD600 = 0.3).

Compared to the control group (Figure 6A), *A. konjac* corms inoculated with *P. aroidearum* alone turned black and decayed significantly (Figure 6B). In comparison, *A. konjac* corm belonging to the GZY0 pretreatment group showed significantly lower morbidity (Figure 6C), and the rot control efficacy reached 45.81% (Table 5).

**Table 5:** Control effect of GZY0 on *P. aroidearum* disease in pot experiments.

**Treatment**	**Disease index**
A	0.00 ± 0.00 c
B	81.33 ± 0.00 a
C	44.07 ± 0.64 b

(A) Root irrigation with water. (B) *P. aroidearum* cell suspension root irrigation. (C) GZY0 cell suspension + *P. aroidearum* cell suspension root irrigation (OD600 = 0.3). Data are means ± standard error, and different small letters indicate significant differences at the 0.05 level.

## 4 Discussion

PGPR are a group of microorganisms that live in the soil or attach to plant roots, promote plant growth, and enhance plant stress tolerance (Tran et al., 2022). PGPR encompass a wide variety of microbial species with diverse functions and play an important role in regulating the rhizosphere microenvironment and promoting plant growth. Thus, they exhibit good application prospects in promoting crop growth and preventing and controlling plant diseases.

In this study, GZY0, a bacterial strain demonstrating antagonism against *P. aroidearum*, was isolated from the rhizospheric soil of *A. konjac* using the solid agar plate confrontation method. Through 16S rRNA sequencing, housekeeping gene *gyrA* sequencing, and ANI analysis, this strain was identified as *B. velezensis*. Notably, *B. velezensis* is a common rhizobacterium, and several *B. velezensis* isolates have demonstrated biocontrol potential against plant pathogens. For instance, *B. velezensis* BS1 isolated from the rhizospheric soil of pepper plants (Shin et al., 2021) can effectively prevent and control anthracnose caused by *Colletotrichum scovillei* in pepper. Meanwhile, *B. velezensis* Bv S3 isolated from the rhizospheric soil of rice seedlings (Jiang et al., 2024) can prevent and control rice seedling blight caused by *F. oxysporum*, and *B. velezensis* isolated from the rhizospheric soil of potato plants (Almasoudi et al., 2024) shows high antagonism against soft rot caused by *P. carotovorum* subsp. *carotovorum* in potato tubers.

In the present study, both the cell suspension and CFS of GZY0 showed significant antagonism against the pathogen *P. aroidearum*. The bacteriostatic zone of GZY0 against *P. aroidearum* measured 15.63 mm, which was larger than the bacteriostatic zones (13 mm and 5–15 mm) observed for Strains TP199 and CAB-L026 against *P. carotovorum* subsp. *Carotovorum*, as reported by Padilla-Gálvez et al. (2021) and Dong et al. (2021), respectively. However, it was smaller than the bacteriostatic zones (2.2, 2.6, and 2.0 cm) observed for Strains 1, 11, and 12 against *P. carotovorum* subsp. *carotovorum*, respectively, in the study by Almasoudi et al. (2024). Subsequently, *in vitro* antagonistic tests and greenhouse assays also verified the biocontrol value of GZY0 against *P. aroidearum*. For instance, the control efficacy of strain GZY0 against *P. aroidearum* on *A. konjac* tissues was higher than that of strain Z10 (20.01%) (Chen, 2013), but lower than that of strain BCP6 (65.65%) (Zhu et al., 2024). Overall, the findings showed that GZY0 provides high biocontrol efficacy against *P. aroidearum* in *A. konjac* tissues, further field trials are required to evaluate its efficacy in controlling soft rot disease in *A. konjac*.

The genomic analysis of GZY0 revealed the presence of 12 gene clusters encoding secondary metabolites, of which five gene clusters (macrolactin H, bacillaene, fengycin, difficidin, and bacilysin) exhibited 100% similarity to the genes in *B. velezensis* FZB42. Meanwhile, one gene cluster (bacillibactin) showed 100% similarity to the genes in *B. subtilis* subsp. *subtilis* str. 168. Macrolactin represents a new class of compounds formed via the cyclization of polyketide chains, and several studies have demonstrated its significant inhibitory effects on various plant pathogens (Yuan et al., 2012; Zhang Y. et al., 2022). Bacillaene, a class of polyketide compounds that inhibit protein synthesis, offers good inhibitory effects against both bacterial and fungal growth. Meanwhile, fengycin inhibits fungal growth by dissolving or disrupting cell membranes (Jacques, 2011). Finally, difficidin and bacilysin act as antimicrobial agents against various bacteria; the former inhibits bacterial protein synthesis and disrupts cell membrane function, while the latter inhibits 6-phosphate glucosamine synthesis and interferes with cell wall formation (Rabbee et al., 2019). As a siderophore antibiotic, bacillibactin demonstrates bactericidal activity against various soil-borne pathogens, including *F. oxysporum* and *P. aroidearum* (Caulier et al., 2018). In addition to these six gene clusters, the gene clusters linked to surfactin and plantazolicin showed 91% similarity with the genes of *B. velezensis* FZB42, which suggested that they may regulate the synthesis of various surfactin and plantazolicin compounds. Among them, surfactin has been shown to act as a potent surfactant molecule, exhibiting antagonistic activity against various pathogenic bacteria (Fazle Rabbee and Baek, 2020). Meanwhile, plantazolicin can stimulate plant growth and inhibit invasion by plant pathogens (Scholz et al., 2011; Mülner et al., 2020). The similarity of Butirosin A/B to *B. circulans* gene clusters was only 7%, suggesting that these gene clusters may synthesize novel bacteriostatic substances, warranting further investigation. Butirosin is an aminoglycoside antibiotic that inhibits *Pseudomonas* and *Salmonella* (Heifetz et al., 1974). Additionally, the gene clusters of GZY0 were not identical to those of *B. velezensis* AMR25 (Ananev et al., 2024), *B. velezensis* 160 (González-León et al., 2024), and *B. velezensis* LS69 (Liu et al., 2017), suggesting that the secondary metabolites secreted by different strains may be unique due to differences in culture conditions. Furthermore, their biocontrol effects with regard to preventing and treating plant diseases may also be distinct. The identification of three other unknown secondary metabolite synthesis gene clusters suggested that GZY0 contains novel antibacterial substances. Comparative genomic analysis indicates that GZY0 possesses a stable core gene set and environmentally adaptable pan-genes. Notably, the 171 unique homologous genes identified in GZY0 highlight its genetic uniqueness and suggest the existence of uncharacterized metabolic pathways or functional features. This genetic distinctiveness provides a potential molecular basis for its unique biocontrol and growth-promoting activities during rhizosphere colonization of *A. konjac*. Further research will focus on characterizing these unique gene clusters to elucidate the mechanisms underlying GZY0′s ability to controlling soft rot disease in *A. konjac*. Hence, our findings pave the way for the further exploration of the biocontrol potential of GZY0 and the discovery of novel bacteriostatic substances.

Many *B. velezensis* strains isolated from the rhizospheric soil have also been shown to promote plant growth. For instance, *B. velezensis* 83, Bv-25, and Ag75 isolated by Balderas-Ruíz et al. (2020), Tian et al. (2022), and Mosela et al. (2022) have been shown to promote the growth of corn, cucumber, and soybean, respectively. Hasan et al. (2022) demonstrated that *B. velezensis* contained genes linked to growth promotion in *Gossypium hirsutum* L. Meanwhile, Shin et al. (2021) reported that *B. velezensis* contained siderophore synthesis genes with good PGP capacity for pepper seedlings. Iqbal et al. (2021) investigated the PGP potential of *B. subtilis* RS10 and found that *B. subtilis* RS10 can promote plant growth by participating in phosphorus solubilization, hydrogen sulfide production, plant hormone synthesis, and siderophore production, suggesting that *B. subtilis* RS10 has immense PGP potential. In this study, *B. velezensis* GZY0 was also found to possess PGP features such as nitrogen fixation, phosphorus solubilization, and siderophore and IAA production capacity. Meanwhile, whole-genome analysis showed that GZY0 possessed PGP-related genes, including nitrogen fixation, IAA synthesis, siderophore, and phosphorus solubilization genes, as well as key genes regulating the production of ABA and salicylic acid. This suggested that strain GZY0 has the potential to generate systemic resistance in plants, laying a theoretical foundation for further in-depth research on the relationship between GZY0 and plant pathogens. Additionally, unknown genes regulating the nitrogen and tryptophan metabolism pathways in GZY0 were identified and need to be explored further.

## 5 Conclusion

In summary, *B. velezensis* GZY0 isolated from the healthy rhizospheric soil of *A. konjac* shows antagonistic activity against various plant pathogens and strong control efficacy against *P. aroidearum*. Additionally, it exhibits good PGP features and has broad application prospects in the biocontrol of plant diseases and promotion of crop growth. GZY0 contains 12 secondary metabolite synthesis clusters and induced systemic resistance-related genes, as well as a number of plant growth-promoting genes, including those regulating nitrogen fixation, phosphorus solubilization, and IAA and siderophore production. Additionally, while GZY0 possesses stable core genes and an open pan-genome, it also harbors a wealth of specific genes that remain to be explored. Thus, this strain has great potential for application during the later stages of agricultural production. Overall, GZY0 may serve as an excellent dual-acting drug–fertilizer strain, enabling the prevention and control of soft rot in *A. konjac*. Future studies should thus focus on the development and application of GZY0 in this domain.

## Data Availability

The BioProject and associated SRA metadata can be accessed at the following URL: https://dataview.ncbi.nlm.nih.gov/object/PRJNA1348301?reviewer=8cs82bngkgoc79peif9thmto1j.
