# Supplementary material for: Isolation and genomic characterization of the plant growth-promoting rhizobacterium GZY0 and its biocontrol potential against soft rot in Amorphophallus konjac
Source: Front Microbiol. 2025 Dec 3;16:1641541. doi: 10.3389/fmicb.2025.1641541 (PMC12708542; doi:10.3389/fmicb.2025.1641541)
Supplement: Supplementary file 1 [file Data_Sheet_1.docx]

Supplementary Material

# Supplementary Figures and Tables


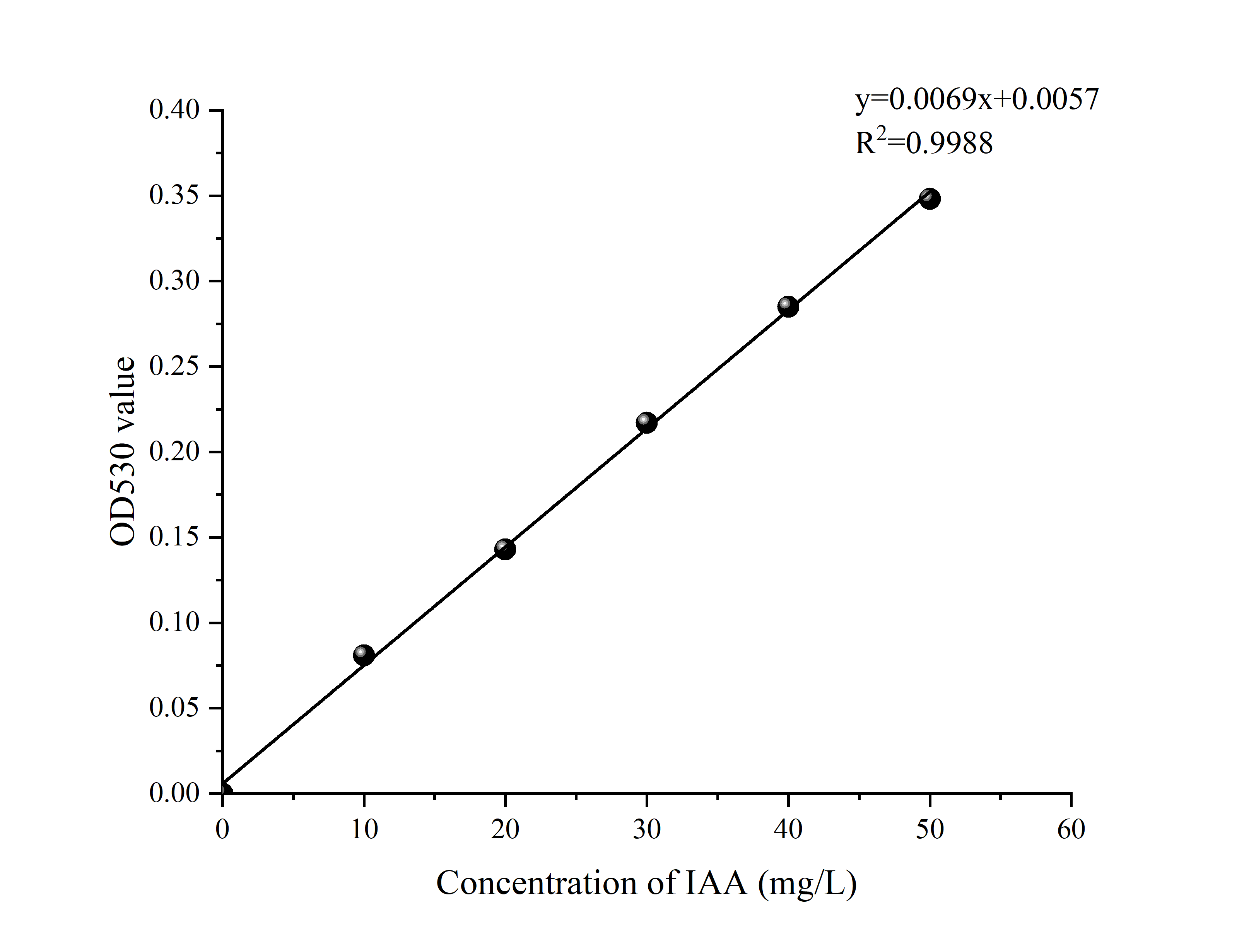


Supplementary Figure 1. IAA standard curve.


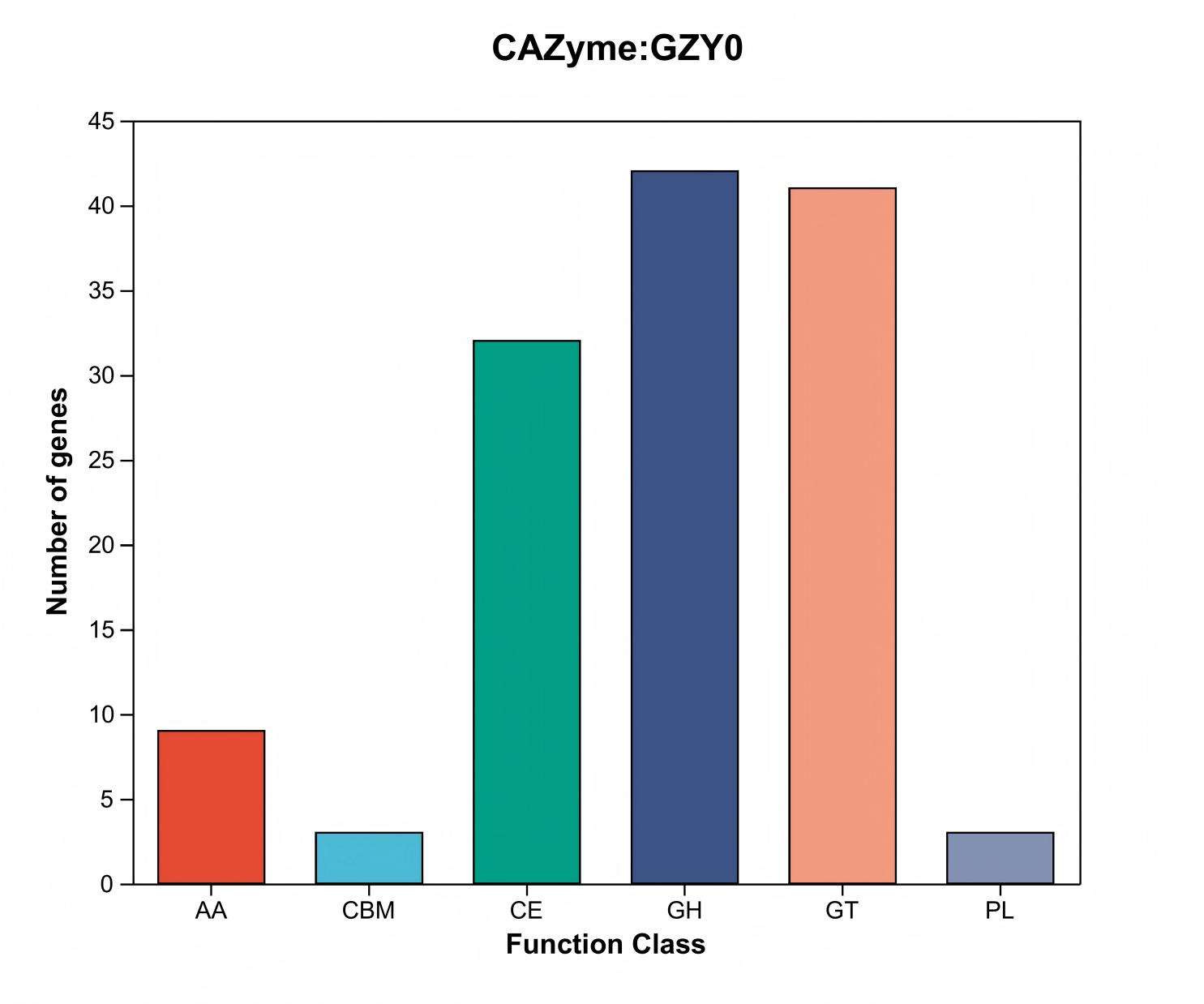


**A**


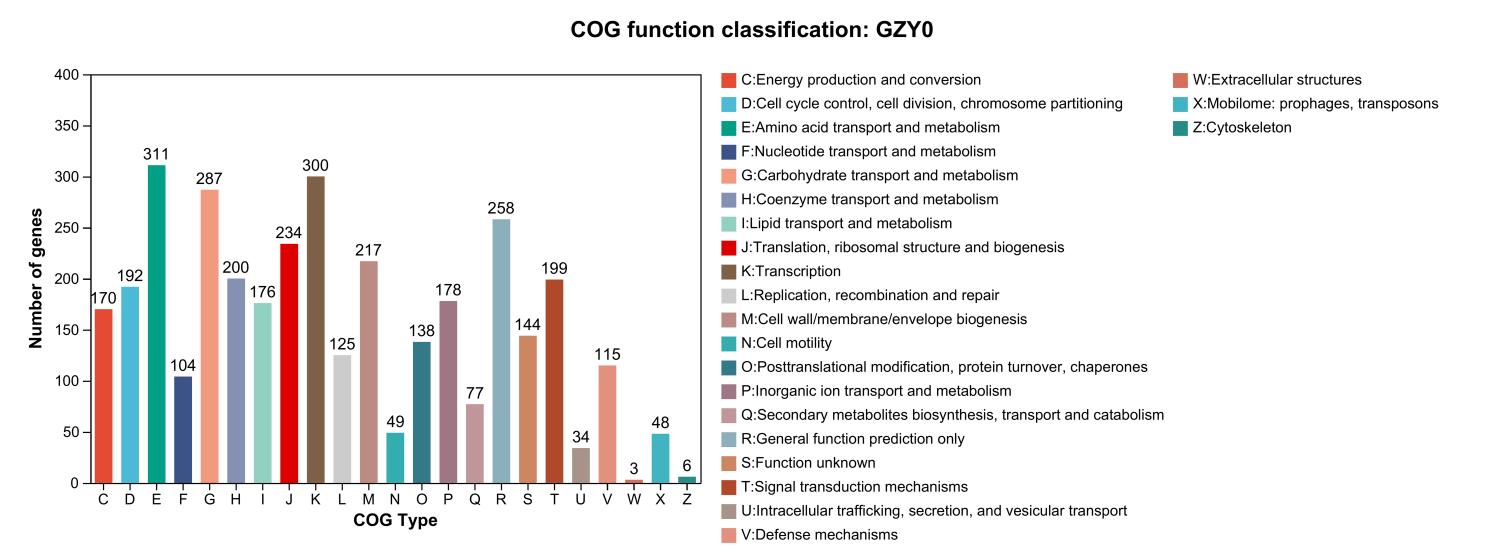


**B**


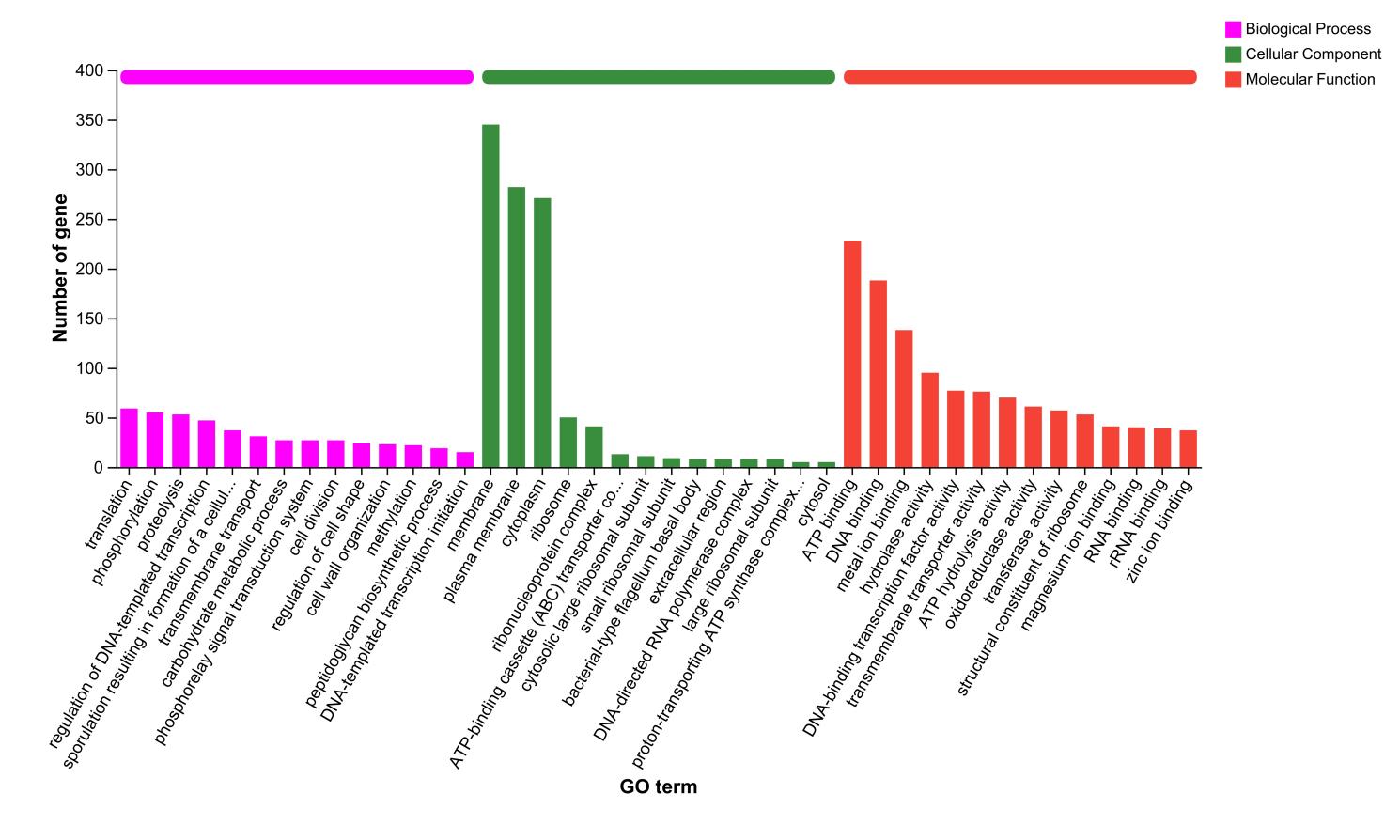


**C**

**Supplementary Figure** 2**.** General genomic features of strain GZY0. **(A)** Carbohydrate active enzyme annotation statistics. **(B)** COG classification statistics histogram. **(C)** GO annotation classification statistics. **(D)** Pathway classification statistics histogram.


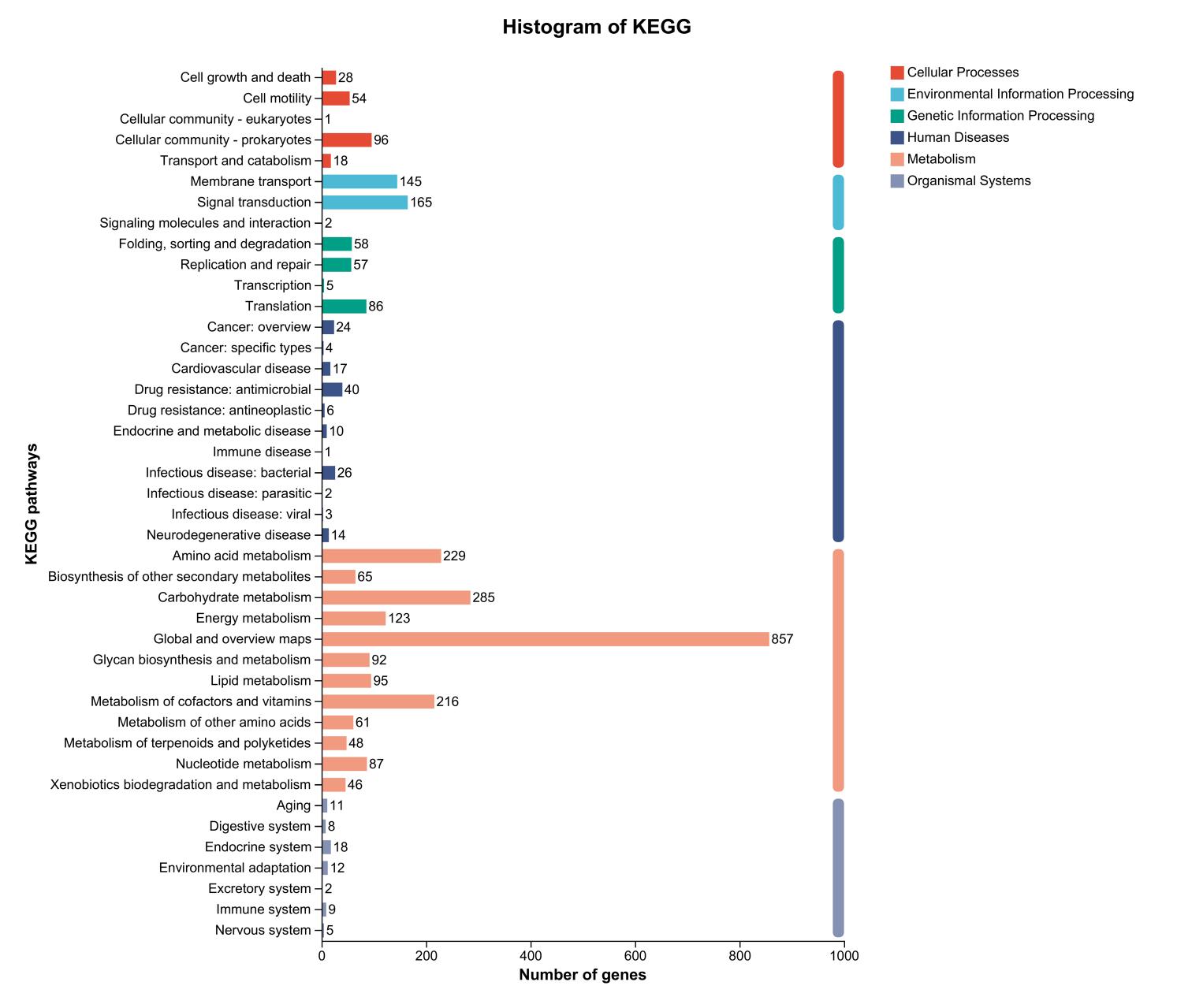


**D**


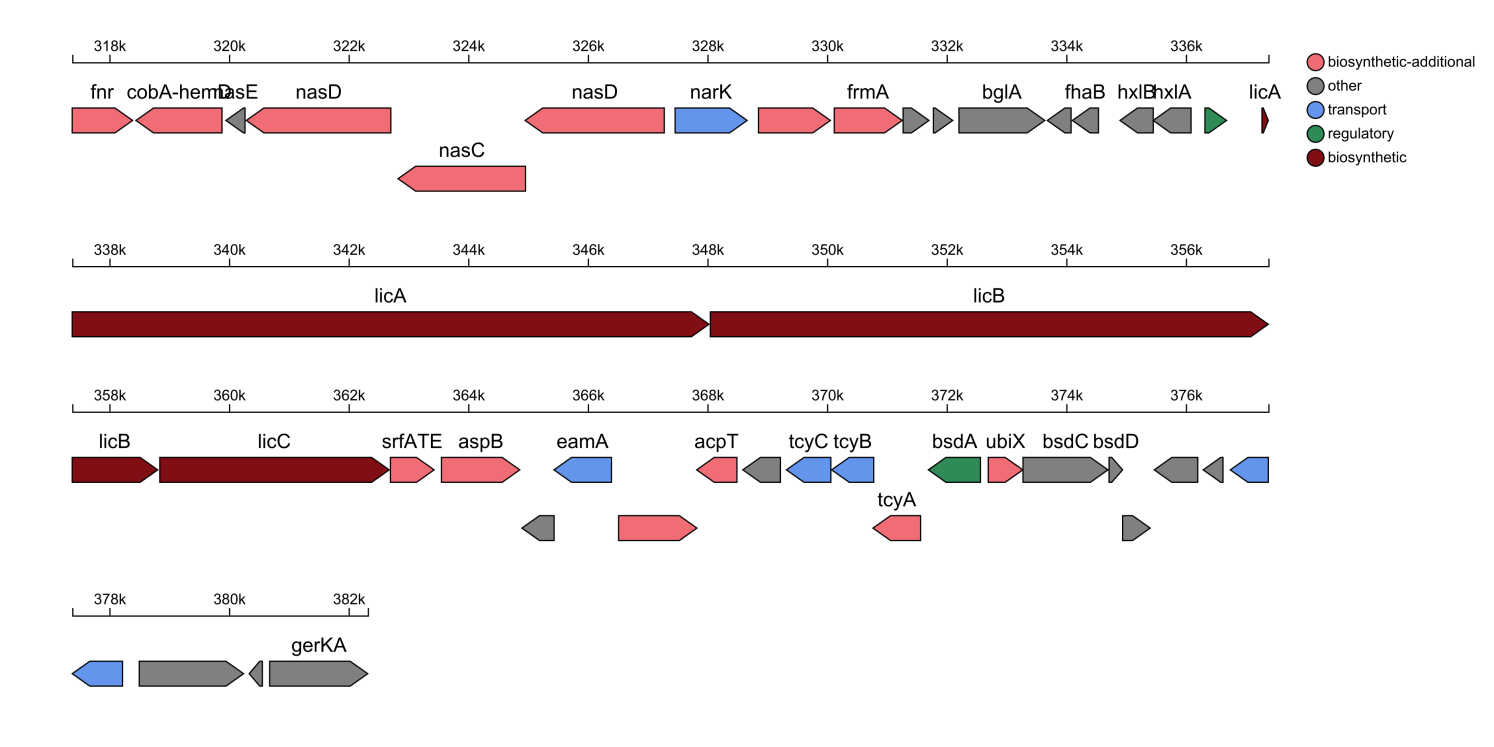


**Supplementary Figure 3.** Linear map of secondary metabolite synthesis gene clusters.

**Supplementary Table 1.**  GZY0 related gene annotation

| **Gene ID** | **KO ID** | **KO Name** | **KO Description** |
| --- | --- | --- | --- |
| gene0343 | K26138 | nasE | nitrite reductase [NAD(P)H] small subunit [EC:1.7.1.4] |
| gene0344  gene0346 | K26139 | nasD | nitrite reductase [NAD(P)H] large subunit [EC:1.7.1.4] |
| gene0345 | K00372 | nasC | assimilatory nitrate reductase catalytic subunit [EC:1.7.99.-] |
| gene0347  gene3682 | K02575 | narK | MFS transporter, NNP family, nitrate/nitrite transporter |
| gene1757 | K01915 | glnA | glutamine synthetase [EC:6.3.1.2] |
| gene1853 | K01743 | - | carbonic anhydrase [EC:4.2.1.1] |
| gene1976 | K00266 | gltD | glutamate synthase (NADPH) small chain [EC:1.4.1.13] |
| gene1977 | K00265 | gltB | glutamate synthase (NADPH) large chain [EC:1.4.1.13] |
| gene2229  gene3735 | K00260 | gudB | glutamate dehydrogenase [EC:1.4.1.2] |
| gene2897  gene3426 | K01673 | cynT | carbonic anhydrase [EC:4.2.1.1] |
| gene3065  gene3066 | K00459 | npd | nitronate monooxygenase [EC:1.13.12.16] |
| gene3675 | K00374 | narI | nitrate reductase gamma subunit [EC:1.7.5.1 1.7.99.-] |
| gene3677 | K00371 | narH | nitrate reductase / nitrite oxidoreductase, beta subunit [EC:1.7.5.1 1.7.99.-] |
| gene3678 | K00370 | narG | nitrate reductase / nitrite oxidoreductase, alpha subunit [EC:1.7.5.1 1.7.99.-] |
| gene3603 | K04751 | glnB | nitrogen regulatory protein P-II 1 |
| gene1353 | K03498 | trkH | trk/ktr system potassium uptake protein |
| gene2795  gene3926  gene2020 | K00128 | - | aldehyde dehydrogenase (NAD+) [EC:1.2.1.3] |
| gene1751  gene2199 | K01609 | trpC | indole-3-glycerol phosphate synthase [EC:4.1.1.48] |
| gene2196 | K01695 | trpA | tryptophan synthase alpha chain [EC:4.2.1.20] |
| gene2197 | K01696 | trpB | tryptophan synthase beta chain [EC:4.2.1.20] |
| gene2198 | K01817 | trpF | phosphoribosylanthranilate isomerase [EC:5.3.1.24] |
| gene2200 | K00766 | trpD | anthranilate phosphoribosyltransferase [EC:2.4.2.18] |
| gene2201 | K01657 | trpE | anthranilate synthase component I [EC:4.1.3.27] |
| gene1149 | K01867 | trpS | tryptophanyl-tRNA synthetase[EC:6.1.1.2] |
| gene2907 | K02552 | menF | menaquinone-specific isochorismate synthase [EC:5.4.4.2] |
| gene3138 | K01252 | entB | bifunctional isochorismate lyase / aryl carrier protein [EC:3.3.2.1 6.3.2.14] |
| gene3139 | K02363 | entE | 2,3-dihydroxybenzoate---[aryl-carrier protein] ligase [EC:6.3.2.14 6.2.1.71] |
| gene3140 | K02361 | entC | isochorismate synthase [EC:5.4.4.2] |
| gene3141 | K00216 | entA | 2,3-dihydro-2,3-dihydroxybenzoate dehydrogenase [EC:1.3.1.28] |
| gene1039 | K01772 | hemH | protoporphyrin/coproporphyrin ferrochelatase [EC:4.98.1.1 4.99.1.9] |
| gene1040 | K00231 | hemY | protoporphyrinogen/coproporphyrinogen III oxidase [EC:1.3.3.4 1.3.3.15] |
| gene1572 | K03794 | sirB | sirohydrochlorin ferrochelatase [EC:4.99.1.4] |
| gene0399 | K25284 | yclN | iron-siderophore transport system permease protein |
| gene0400 | K25283 | yclO | iron-siderophore transport system permease protein |
| gene0401 | K25285 | yclP | iron-siderophore transport system ATP-binding protein [EC:7.2.2.-] |
| gene0402 | K25282 | yclQ | iron-siderophore transport system substrate-binding protein |
| gene1234 | K02069 | fetB | UDP-glucose/iron transport system permease protein |
| gene1235 | K02068 | fetA | UDP-glucose/iron transport system ATP-binding protein |
| gene3359 | K00927 | pgk | phosphoglycerate kinase [EC:2.7.2.3] |
| gene0019 | K00865 | glxK | glycerate 2-kinase [EC:2.7.1.165] |
| gene1987  gene2239 | K00058 | serA | D-3-phosphoglycerate dehydrogenase / 2-oxoglutarate reductase [EC:1.1.1.95 1.1.1.399] |
| gene3357 | K15633 | gpmI | 2,3-bisphosphoglycerate-independent phosphoglycerate mutase [EC:5.4.2.12] |
| gene1312 | K07404 | pgl | 6-phosphogluconolactonase [EC:3.1.1.31] |
| gene1909 | K00874 | kdgK | 2-dehydro-3-deoxygluconokinase [EC:2.7.1.45] |
| gene3377 | K25031 | gntK | gluconokinase [EC:2.7.1.12] |
| gene3913 | K03338 | iolC | 5-dehydro-2-deoxygluconokinase [EC:2.7.1.92] |
| gene1911 | K01625 | eda | 2-dehydro-3-deoxyphosphogluconate aldolase / (4S)-4-hydroxy-2-oxoglutarate aldolase [EC:4.1.2.14 4.1.3.42] |
| gene2335 | K00033 | gntZ | 6-phosphogluconate dehydrogenase [EC:1.1.1.44 1.1.1.343] |
| gene3427 | K00090 | ghrB | glyoxylate/hydroxypyruvate/2-ketogluconate reductase [EC:1.1.1.79 1.1.1.81 1.1.1.215] |
| gene3906 | K03339 | iolJ | 6-phospho-5-dehydro-2-deoxy-D-gluconate aldolase [EC:4.1.2.29] |
| gene0322 | K00016 | ldh | L-lactate dehydrogenase [EC:1.1.1.27] |
| gene2660 | K01653 | ilvH | acetolactate synthase I/III small subunit [EC:2.2.1.6] |
| gene2661  gene3553 | K01652 | ilvB | acetolactate synthase I/II/III large subunit [EC:2.2.1.6] |
| gene3552 | K01575 | alsD | acetolactate decarboxylase [EC:4.1.1.5] |
| gene2182  gene2949  gene3267 | K01569 | oxdD | oxalate decarboxylase [EC:4.1.1.2] |
| gene3244  gene3244 | K01679 | fumC | fumarate hydratase, class II [EC:4.2.1.2] |
| gene2681 | K00239 | sdhA | succinate dehydrogenase flavoprotein subunit [EC:1.3.5.1] |
| gene2773 | K01940 | argG | argininosuccinate synthase [EC:6.3.4.5] |
| gene0410 | K00135 | gabD | succinate-semialdehyde dehydrogenase / glutarate-semialdehyde dehydrogenase [EC:1.2.1.16 1.2.1.79 1.2.1.20] |
| gene0671 | K01756 | purB | adenylosuccinate lyase [EC:4.3.2.2] |
| gene2680 | K00240 | sdhB | succinate dehydrogenase iron-sulfur subunit [EC:1.3.5.1] |
| gene2682 | K00241 | sdhC | succinate dehydrogenase cytochrome b subunit |
| gene2772 | K01755 | argH | argininosuccinate lyase [EC:4.3.2.1] |
| gene3980 | K01939 | purA | adenylosuccinate synthase [EC:6.3.4.4] |
| gene0332 | K15652 | asbF | 3-dehydroshikimate dehydratase [EC:4.2.1.118] |
| gene0334 | K00891 | aroK | shikimate kinase [EC:2.7.1.71] |
| gene0801 | K05887 | ydiB | quinate/shikimate dehydrogenase [EC:1.1.1.282] |
| gene2180  gene2193 | K00800 | aroA | 3-phosphoshikimate 1-carboxyvinyltransferase [EC:2.5.1.19] |
| gene2514 | K00014 | aroE | shikimate dehydrogenase [EC:1.1.1.25] |
| gene0409 | K07250 | gabT | 4-aminobutyrate aminotransferase / (S)-3-amino-2-methylpropionate transaminase / 5-aminovalerate transaminase [EC:2.6.1.19 2.6.1.22 2.6.1.48] |
| gene1404 | K00020 | mmsB | 3-hydroxyisobutyrate dehydrogenase [EC:1.1.1.31] |
| gene1935  gene4017 | K00019 | bdhA | 3-hydroxybutyrate dehydrogenase [EC:1.1.1.30] |
| gene2354 | K00929 | buk | butyrate kinase [EC:2.7.2.7] |
| gene0776 | K11639 | citM | Mg2+/citrate complex secondary transporter |
| gene2747 | K00031 | icd | isocitrate dehydrogenase [EC:1.1.1.42] |
| gene0970  gene2361  gene2748 | K01647 | gltA | citrate synthase [EC:2.3.3.1] |
| gene2359 | K03417 | prpB | methylisocitrate lyase [EC:4.1.3.30] |
| gene2360 | K01720 | prpD | 2-methylcitrate dehydratase [EC:4.2.1.79] |
| gene2070 | K01035 | atoA | acetate CoA/acetoacetate CoA-transferase beta subunit [EC:2.8.3.8 2.8.3.9] |
| gene2071 | K01034 | atoD | acetate CoA/acetoacetate CoA-transferase alpha subunit [EC:2.8.3.8 2.8.3.9] |
| gene0258 | K00483 | hpaB | 4-hydroxyphenylacetate 3-monooxygenase [EC:1.14.14.9] |
| gene2775 | K00925 | ackA | acetate kinase [EC:2.7.2.1] |
| gene3231 | K01574 | adc | acetoacetate decarboxylase [EC:4.1.1.4] |
| gene2287  gene2761  gene2829  gene3656 | K00027 | sfcA | malate dehydrogenase (oxaloacetate-decarboxylating) [EC:1.1.1.38] |
| gene3843 | K11616 | maeN | malate:Na+ symporter |
| gene0594  gene0595 | K07246  K07246 | ttuC | tartrate dehydrogenase/decarboxylase / D-malate dehydrogenase [EC:1.1.1.93 4.1.1.73 1.1.1.83] |
| gene2655 | K01704 | leuD | 3-isopropylmalate/(R)-2-methylmalate dehydratase small subunit [EC:4.2.1.33 4.2.1.35] |
| gene2656 | K01703 | leuC | 3-isopropylmalate/(R)-2-methylmalate dehydratase large subunit [EC:4.2.1.33 4.2.1.35] |
| gene2657 | K00052 | leuB | 3-isopropylmalate dehydrogenase [EC:1.1.1.85] |
| gene2658 | K01649 | leuA | 2-isopropylmalate synthase [EC:2.3.3.13] |
| gene2746 | K00024 | mdh | malate dehydrogenase [EC:1.1.1.37] |
| gene1400 | K08483 | ptsI | phosphoenolpyruvate-protein phosphotransferase (PTS system enzyme I) [EC:2.7.3.9] |
| gene1473 | K00161 | pdhA | pyruvate dehydrogenase E1 component subunit alpha [EC:1.2.4.1] |
| gene1474 | K00162 | pdhB | pyruvate dehydrogenase E1 component subunit beta [EC:1.2.4.1] |
| gene0451 | K00158 | spxB | pyruvate oxidase [EC:1.2.3.3] |
| gene0827  gene1475 | K00627 | aceF | pyruvate dehydrogenase E2 component (dihydrolipoyllysine-residue acetyltransferase) [EC:2.3.1.12] |
| gene0952 | K03851 | tpa | taurine-pyruvate aminotransferase [EC:2.6.1.77] |
| gene1095 | K16164 | - | acylpyruvate hydrolase [EC:3.7.1.5] |
| gene1498 | K01958 | pyc | pyruvate carboxylase [EC:6.4.1.1] |
| gene2072 | K12256 | spuC | putrescine---pyruvate transaminase [EC:2.6.1.113] |
| gene2757 | K00873 | pyk | pyruvate kinase [EC:2.7.1.40] |
| gene2884 | K01610 | pckA | phosphoenolpyruvate carboxykinase (ATP) [EC:4.1.1.49] |
| gene2744  gene3438  gene0394 | K07636 | phoR | two-component system, OmpR family, phosphate regulon sensor histidine kinase PhoR [EC:2.7.13.3] |
| gene0286 | K01113 | phoD | alkaline phosphatase D [EC:3.1.3.1] [EC:3.1.3.1] |
| gene2745 | K07658 | phoP | two-component system, OmpR family, alkaline phosphatase synthesis response regulator PhoP |
| gene0967  gene1346 | K01077 | phoA | alkaline phosphatase [EC:3.1.3.1] [EC:3.1.3.1] |
| gene2448 | K02040 | pstS | phosphate transport system substrate-binding protein |
| gene2446 | K02038 | pstA | phosphate transport system permease protein |
| gene2447 | K02037 | pstC | phosphate transport system permease protein |
| gene3536  gene0968  gene1384 | K19224 | lytE | peptidoglycan DL-endopeptidase LytE [EC:3.4.-.-] |
| gene1310 | K20742 | ykfC | gamma-D-glutamyl-L-lysine dipeptidyl-peptidase [EC:3.4.14.13] |
| gene2700 | K01890 | pheT | phenylalanyl-tRNA synthetase beta chain [EC:6.1.1.20] |
| gene2701 | K01889 | pheS | phenylalanyl-tRNA synthetase alpha chain [EC:6.1.1.20] |

Note: “-” indicates that it has not been commented on.
